# Poor healthy lifestyle and life’s essential 8 are associated with higher risk of new-onset migraine: a prospective cohort study

**DOI:** 10.1186/s10194-024-01785-4

**Published:** 2024-05-17

**Authors:** Yuexiu Lei, Lili Zhang, Zhengming Shan, Quan Gan, Qingfang Xie, Ying Huang, Wen Yan, Zheman Xiao

**Affiliations:** Department of Neurology, Renmin Hospital of Wuhan University, 99 Zhang Zhidong Road, Wuchang District, Wuhan, Hubei Province 430060 China

**Keywords:** Healthy lifestyle, Life’s essential 8, Migraine, Lifestyle factors

## Abstract

**Background:**

Lifestyle are closely related to migraine. However, there is a lack of studies investigating the association between Healthy lifestyle or Life’s Essential 8 (LE8) and the risk of migraine. The objective of this research was to investigate the relationship between Healthy lifestyle scores and Life’s essential 8 scores, and migraine.

**Methods:**

332,895 UK Biobank participants without migraine were included. Healthy lifestyle were assessed using seven lifestyle factors, and categorized as poor, intermediate, or ideal. LE8, based on the American Heart Association (AHA) Guidelines for Cardiovascular Health (CVH), consist of eight indicators classified as low, moderate, or high CVH. The Cox proportional hazard model was employed to examine the association between Healthy lifestyle scores, LE8 scores, and migraine, with calculations for population-attributable fraction (PAF) and cumulative incidence.

**Results:**

During a median follow-up of 13.58 years, participants in intermediate (HR: 0.91; 95% CI: 0.85, 0.99) or ideal category of Healthy lifestyle (HR: 0.81; 95% CI: 0.73, 0.91) significantly reduced migraine risk compared to the poor category. Similarly, high CVH (HR: 0.73; 95% CI: 0.58, 0.92) also lowered migraine risk, while moderate CVH (HR: 0.93; 95% CI: 0.85, 1.02) did not show a difference compared to low CVH. If all individuals adhered to higher categories of Healthy lifestyle and LE8, approximately 11.38% and 22.05% of migraine cases could be prevented. Among individual lifestyle factors, maintaining an ideal body mass index (BMI), physical activity, sleep duration, sleep pattern, and sedentary time were associated with substantial reductions in migraine risk, by 5.65%, 0.81%, 10.16%, 16.39%, and 6.57%, respectively.

**Conclusion:**

Our study provides evidence that poor Healthy lifestyle and Life’s Essential 8 are associated with higher risk of new-onset migraine.

**Supplementary Information:**

The online version contains supplementary material available at 10.1186/s10194-024-01785-4.

## Background

Migraine, a neurological condition, is identified by recurring, pulsating headache of varying degrees of intensity. It is often accompanied by symptoms like nausea, vomiting, and increased sensitivity to light and sound [1]. Globally, approximately 1.04 billion people suffer from migraine, positioning it as the second most prevalent reason for disability. Moreover, the lifetime prevalence of migraine in women is approximately twice that of men [2, 3]. The treatment and management of migraine typically entail a combination of pharmacological interventions and modifications to one’s lifestyle.

In recent years, a growing body of evidence of the association between lifestyle factors and migraine. These studies have identified a potential association between specific dietary habits, such as excessive intake of caffeine, alcohol, and chocolate, and the occurrence of migraine attacks [4, 5]. Additionally, poor sleep quality, irregular sleep pattern, and inadequate physical exercise have been recognized as potential triggers for migraine [6–9]. Nevertheless, the majority of investigations have predominantly employed retrospective or cross-sectional study designs, thereby hindering the ability to establish causal relationships [10, 11]. Additionally, a comprehensive evaluation of the influence of lifestyle on migraine has been largely overlooked in most studies. Consequently, additional long-term, large-scale studies as well as a consideration of integrated lifestyles are imperative to delve deeper into the association between lifestyle factors and migraine.

Healthy lifestyle is a comprehensive approach that takes into account diverse lifestyle factors. An increasing number of studies are employing this approach to examine the link between lifestyle and diseases [12, 13]. Life’s essential 8 (LE8), a set of health factors and behaviors crucial for maintaining cardiovascular health (CVH), has been identified by the American Heart Association [14]. The factors include diet, physical activity, smoking, sleep, weight, and blood lipids, blood glucose, and blood pressure. The coexistence of migraine and specific cardiovascular diseases has been observed [15, 16], indicating that strategies aimed at protecting the heart according to the LE8 may also have potential in preventing migraine. However, the precise understanding of how Healthy lifestyle and LE8 influence migraine remains unclear.

Therefore, the current study aimed to explore the relationship between Healthy lifestyle scores, LE8 scores, and migraine. Concurrently, the study aims to identify key lifestyle factors that offer valuable insights for the primary prevention of migraine.

## Methods

### Population and study design

This study utilized data from the UK Biobank (Application Number: 143,136), a large, ongoing prospective cohort study. Between 2006 and 2010, the UK Biobank collected a substantial dataset from more than 500,000 individuals aged 37 to 73 years across the UK. Through touchscreen questionnaires, physical examinations, and collection of biological samples, participants furnished sociodemographic, lifestyle, and health-related details. The UK Biobank also provides participants with health outcome information by establishing connections with diverse health-related records. Detailed data on the UK Biobank is available on the internet at http//www.ukbiobank.ac.uk, in addition to being accessible on published articles [17, 18].

**Fig. 1:**
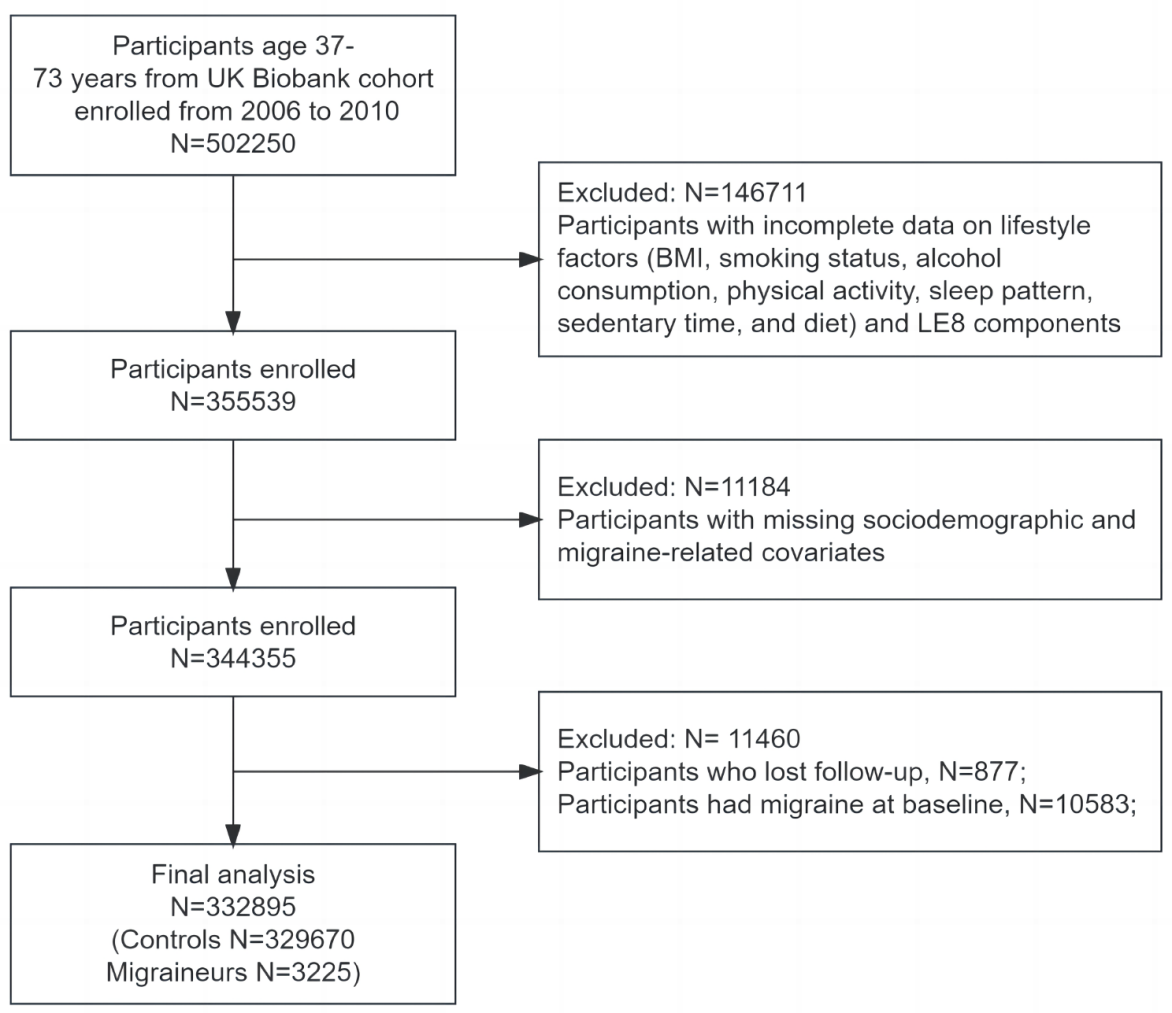
Flowchart of the study. BMI = body mass index; LE8 = life’s essential 8

Initially, we excluded participants from our study who had incomplete data on lifestyle factors (including BMI, smoking status, alcohol consumption, physical activity, sleep pattern, sedentary time, and diet) and LE8 components. Subsequently, participants with missing sociodemographic and migraine-related covariates were also excluded. Furthermore, participants lost to follow-up and those with migraine at baseline were finally excluded. Ultimately, the analysis comprised a total of 332,895 participants (Fig. 1).

Approval for the UK Biobank was granted by the North West Research Ethics Committee under reference number 06/MRE08/65. All participants gave written informed consent prior to enrollment.

### Healthy lifestyle scores

To determine Healthy lifestyle scores, we chose seven modifiable well-established lifestyle factors, encompassing BMI, smoking status, alcohol consumption, physical activity, diet, sleep pattern and sedentary time. Further information of each lifestyle factor can be accessed in Table S1.

The calculation of Body Mass Index (BMI) involved dividing the weight of a person (measured in kilograms) by the square of their height (measured in meters). A BMI below 25 kg/m^2^ was generally considered to be ideal. Smoking status was classified into three categories: never smoked, former smoker, and current smoker, with never smoking being the ideal. Alcohol consumption was calculated by summing the intake of various alcoholic beverages, including wine (red, white, fortified), champagne, beer, cider, spirits, and other alcoholic drinks. In the United Kingdom, the recommended alcohol consumption for women was 0-≤14 g per day, while for men it was 0-≤28 g per day, which was considered to be ideal [13, 19, 20]. Physical activity was evaluated by self-reported measures of the weekly duration of moderate and vigorous physical activity. The attainment or surpassing of 150 min of moderate physical activity, 75 min of vigorous physical activity, or a cumulative total of 150 min of physical activity was defined as ideal [21]. Based on previous studies from UK Biobank [12, 22], we employed a modified dietary score (Table S2) that aligns with the Americanized Mediterranean dietary pattern endorsed by the AHA. The attainment of a score of 5 was deemed ideal in terms of the dietary score. A healthy sleep pattern (Table S3) was formulated by considering five sleep factors, assigning a score of 1 for low risk and 0 for any other condition, resulting in a cumulative score of 5. A total score of 4 or higher indicates the presence of a healthy sleep pattern [23]. The quantification of sedentary time involved the summation of daily durations dedicated to television watching and computer using, wherein an ideal threshold was established at less than 4 h in total [24].

Participants were given a score of 1 if they meet the ideal criteria for each lifestyle factor, and a score of 0 if they did not. The cumulative score for all lifestyle factors was then calculated to determine the Healthy lifestyle scores. Subsequently, Healthy lifestyle scores were classified as ideal (5 or more points), intermediate (3 or 4 points), or poor (0 to 2 points) Healthy lifestyle.

### Life’s essential 8 scores

According to the AHA’s construct of cardiovascular health [14], LE8 scores were made up by eight component metrics, including diet, physical activity, nicotine exposure, sleep, BMI, blood lipids, blood glucose, and blood pressure.

Healthy lifestyle scores section provides an overview of the methodologies employed to BMI, diet, physical activity and smoking status. Nicotine exposure was ascertained by considering both the individual’s smoking status and their exposure to secondhand smoke. The evaluation of secondhand smoke exposure involved inquiring whether anyone in the participant’s household engages in smoking. Sleep duration was recorded by posing the question, “Approximately how many hours of sleep do you typically obtain within a 24-hour period?” Non-high-density lipoprotein (non-HDL) cholesterol, which is calculated by subtracting high-density lipoprotein (HDL) cholesterol from total cholesterol, was used as the primary indicator for blood lipids. Blood glucose levels were evaluated through the utilization of glycated hemoglobin (HbA1c) and consideration of diabetes history. The average of two consecutive measurements was used to determine the systolic and diastolic blood pressure. Furthermore, medication records were scrutinized to ascertain the utilization of cholesterol-lowering and blood pressure medications.

LE8 scores involved eight components, each ranging from 0 to 100. The overall LE8 scores were determined by averaging the scores of these components. As per the guidelines provided by the American Heart Association (AHA) [14], the overall LE8 scores were categorized into three groups: Low CVH (0–49), Moderate CVH (50–79), and High CVH (80–100). Table S4 contain additional comprehensive information on LE8.

### Ascertainment of outcome

The diagnosis of migraine was determined using data obtained from the Tenth Revision of the International Classification of Diseases (ICD10), specifically through hospital admissions and self-report. Migraine was defined by the ICD10, with the data field 41,270 code G43 and/or self-reported with the data field 20,002 code 1265. This study conducted a follow-up on participants who did not have migraine at baseline. The duration of follow-up was determined by measuring the time elapsed from the baseline assessment until the occurrence of migraine diagnosis, death, or the end of the follow-up period (October 31, 2022 for England, August 31, 2022 for Scotland, and May 31, 2022 for Wales), whichever occurred first.

### Ascertainment of covariates

The potential covariates in this study were obtained from questionnaires at baseline. Age, sex and Townsend Deprivation Index (determined by their postcode and indicating higher scores for more deprivation) that were known before arrival at the Assessment Centre. Ethnicity and education were classified as binary variables, with ethnicity categorized as White and Non-White (mixed, Asian or Asian British, black or black British, Chinese, and other), and education categorized as higher degree (college or university degree or other professional qualifications) and other degree (A levels, AS levels, O levels, GCSEs, NVQ or HND or HNC, and other). Average total household income was collected by asking ‘What is the average total income received by your household before tax?’ and was characterized as: £; less than 18 000, 18 000–30 999, 31 000–51 999, 52 000–100 000, greater than 100 000 and Unknown. In addition, a range of medical conditions including vascular/heart problems, cancer and other serious medical condition/disability were also evaluated.

### Statistical analysis

Baseline characteristics of all participants was using analysis of variance (ANOVA), Kruskal-Wallis test, and chi-square test. Continuous variables were expressed as mean ± standard deviation (SD) if they satisfied normal distribution, otherwise as median [interquartile range (IQR)]. Categorical variables were expressed as percentages (%).

The Cox proportional hazards model to evaluate the potential association between migraine, scores in Healthy lifestyle and LE8, and individual lifestyle factors. Proportional hazards testing was performed using Schoenfeld residuals to ensure the validity of the analysis. The data were stratified based on age (> 55 and < = 55), and three distinct models were constructed in the multivariable analysis. In the analysis, Model 1 was initially modified by taking into account sex and ethnicity. Subsequently, Model 2 was underwent additional adjustments for income, education, and Townsend deprivation index. Model 3 incorporated additional adjustments for baseline cardiovascular diseases, cancer, and other serious diseases. Collinearity between covariates was tested using the variance inflation factor (VIF), and all covariates were found to be acceptable (all VIF were < 5). For detailed results, please refer to Tables S5 and S6. To investigate the dose-response effects, restricted cubic splines were utilized for continuous variables. Additionally, the cumulative incidence of migraine during the follow-up period was computed for participants in Healthy lifestyle categories (poor, intermediate, ideal Healthy lifestyle) and LE8 categories (low, moderate, high CVH), employing Kaplan-Meier survival curves. In theory, the incidence of migraine would decrease if all participants adhered to the low-risk lifestyle. To estimate the population attributable fraction (PAF), assuming a causal relationship between lifestyle and migraine risk, the R package AF was utilized.

Stratified analyses and interactions were conducted to assess the impact of Healthy lifestyle categories (per category increment) and LE8 categories (per category increment) on the incidence of migraine using covariates. These covariates included age (< 55 years and ≥ 55 years), sex (female or male), ethnicity (white or non-white), Townsend deprivation index (quintiles 1, quintiles 2–4, quintile 5), education (higher education or lower than higher education), average household income (£; less than 18,000, 18,000–30,999, 31,000–51,999, 52,000–100,000, greater than 100,000, and unknown), baseline cardiovascular disease (yes or no), baseline cancer (yes or no), and baseline other serious diseases (yes or no).

To ensure the dependability of our results, we conducted three sensitivity analyses. Initially, we employed the chained equation algorithm to conduct multiple imputation for missing data in all exposure variables and covariates, thereby evaluating the influence of missing values. Secondly, for further sensitivity analysis, we excluded individuals who developed migraine within 2 years. Lastly, at the outset of the study, we excluded participants with other types of headaches to mitigate the potential for reverse causation.

The statistical analyses were conducted using R software (version 4.3.2, http://www.R-project.org). A two-tailed *p*-value below 0.05 was deemed statistically significant for evaluating disparities.

## Results

### Baseline characteristics of participants

The study included 332,895 participants (Fig. 1), with their baseline characteristics, categorized by the occurrence of migraine, presented in Table 1. During the follow-up period, with a median age of 58.00 years, including 47.2% males, a total of 3,225 individuals were identified as experiencing migraine. Compared to individuals without migraine, those with migraine were younger, had a higher proportion of females (approximately 2.5 times more than males), were more likely to be white, had a higher prevalence of poverty, lower educational achievements, and experienced a higher incidence of previous cardiovascular diseases, cancer, or other severe diseases. On the other hand, non-migraine participants demonstrated better scores in terms of Healthy lifestyle and LE8.

**Table 1:** Baseline characteristics of study population

	Overall	Migraine	Without Migraine	*P* value
Number of participants (%)	332,895(100.0)	3225 (0.9)	329,670 (99.1)	
Age, years	58.00 [50.00, 63.00]	57.00 [49.00, 63.00]	58.00 [50.00, 63.00]	< 0.001***
Men (%)	157,274 (47.2)	913 (28.3)	156,361 (47.4)	< 0.001***
Ethnicity, White (%)	317,419 (95.4)	3051 (94.6)	314,368 (95.4)	0.048*
Townsend deprivation index	-2.23 [-3.68, 0.30]	-1.73 [-3.44, 1.26]	-2.24 [-3.69, 0.29]	< 0.001***
Income (%)				< 0.001***
Less than 18,000	62,282 (18.7)	731 (22.7)	61,551 (18.7)	
18,000 to 30,999	73,627 (22.1)	716 (22.2)	72,911 (22.1)	
31,000 to 51,999	76,922 (23.1)	674 (20.9)	76,248 (23.1)	
52,000 to 100,000	61,030 (18.3)	476 (14.8)	60,554 (18.4)	
Greater than 100,000	16,468 (4.9)	115 (3.6)	16,353 (5.0)	
Unknown	42,566 (12.8)	513 (15.9)	42,053 (12.8)	
Higher degree (%)	161,017 (48.4)	1489 (46.2)	159,528 (48.4)	0.013*
CVD (%)	96,744 (29.1)	1039 (32.2)	95,705 (29.0)	< 0.001***
Cancer (%)	25,205 (7.6)	282 (8.7)	24,923 (7.6)	0.013*
OSD (%)	66,028 (19.8)	938 (29.1)	65,090 (19.7)	< 0.001***
**Individual lifestyle factors**				
BMI, kg/m^2^	26.67 [24.11, 29.77]	26.89 [24.19, 30.29]	26.67 [24.11, 29.76]	0.002**
Smoking status (%)				0.686
Current	34,020 (10.2)	331 (10.3)	33,689 (10.2)	
Never	181,909 (54.6)	1784 (55.3)	180,125 (54.6)	
Previous	116,966 (35.1)	1110 (34.4)	115,856 (35.1)	
Alcohol, g/day	11.66 [2.72, 24.23]	5.83 [0.91, 16.46]	11.66 [2.72, 24.23]	< 0.001***
Moderate physical activity, min/week	120.00 [30.00, 300.00]	120.00 [30.00, 315.00]	120.00 [30.00, 300.00]	0.659
Vigorous physical activity, min/week	30.00 [0.00, 120.00]	20.00 [0.00, 105.00]	30.00 [0.00, 120.00]	< 0.001***
Diet score	3.00 [2.00, 4.00]	3.00 [2.00, 4.00]	3.00 [2.00, 4.00]	< 0.001***
Sleep duration, h/day	7.00 [7.00, 8.00]	7.00 [6.00, 8.00]	7.00 [7.00, 8.00]	< 0.001***
Sleep score	3.00 [3.00, 4.00]	3.00 [2.00, 4.00]	3.00 [3.00, 4.00]	< 0.001***
Sedentary time, h/day	3.50 [2.50, 5.00]	4.00 [2.50, 5.00]	3.50 [2.50, 5.00]	< 0.001***
Non–HDL cholesterol, mg/dl	161.76 [134.61, 190.72]	162.99 [134.92, 190.84]	161.76 [134.61, 190.72]	0.213
HbA1c (%)	5.37 [5.14, 5.61]	5.38 [5.16, 5.61]	5.37 [5.14, 5.61]	0.414
SBP, mmHg	136.50 [124.50, 149.50]	134.00 [122.00, 147.00]	136.50 [124.50, 149.50]	< 0.001***
DBP, mmHg	82.00 [75.00, 89.00]	81.50 [74.50, 88.00]	82.00 [74.50, 88.00]	< 0.001***
**Healthy lifestyle**				
Healthy lifestyle scores	3.00 [2.00, 4.00]	3.00 [2.00, 4.00]	3.00 [2.00, 4.00]	0.002**
Healthy lifestyle categories				0.003**
Poor ( < = 2)	120,021 (36.1)	1237 (38.4)	118,784 (36.0)	
Intermediate (3–4)	157,356 (47.3)	1509 (46.8)	155,847 (47.3)	
Ideal (≥ 5)	55,518 (16.7)	479 (14.9)	55,039 (16.7)	
**Life’s essential 8**				
LE8 scores	60.62 [53.12, 67.50]	60.00 [52.50, 67.50]	60.62 [53.12, 67.50]	0.008**
LE8 categories				
Low CVH (< 49)	54,530 (16.4)	599 (18.6)	53,931 (16.4)	0.002**
Moderate CVH (50–79)	268,112 (80.5)	2537 (78.7)	265,575 (80.6)	
High CVH (≥ 80)	10,253 (3.1)	89 (2.8)	10,164 (3.1)	

*Abbreviation* CVD, cardiovascular diseases; OSD, other serious diseases; BMI, body mass index; SBP, systolic blood pressure; DBP, diastolic blood pressure

*Data are median [interquartile range (IQR)] for continuous variables or number (%) for categorical variables

*P values were calculated using a Kruskal-Wallis test for continuous variables and a chi-square test for categorical variables, comparing individuals with migraine to those without migraine. Bold values indicate significance, with a *P* value < 0.05. *P < 0.05, **P < 0.01, ***P < 0.001

### Healthy lifestyle scores and migraine

**Fig. 2:**
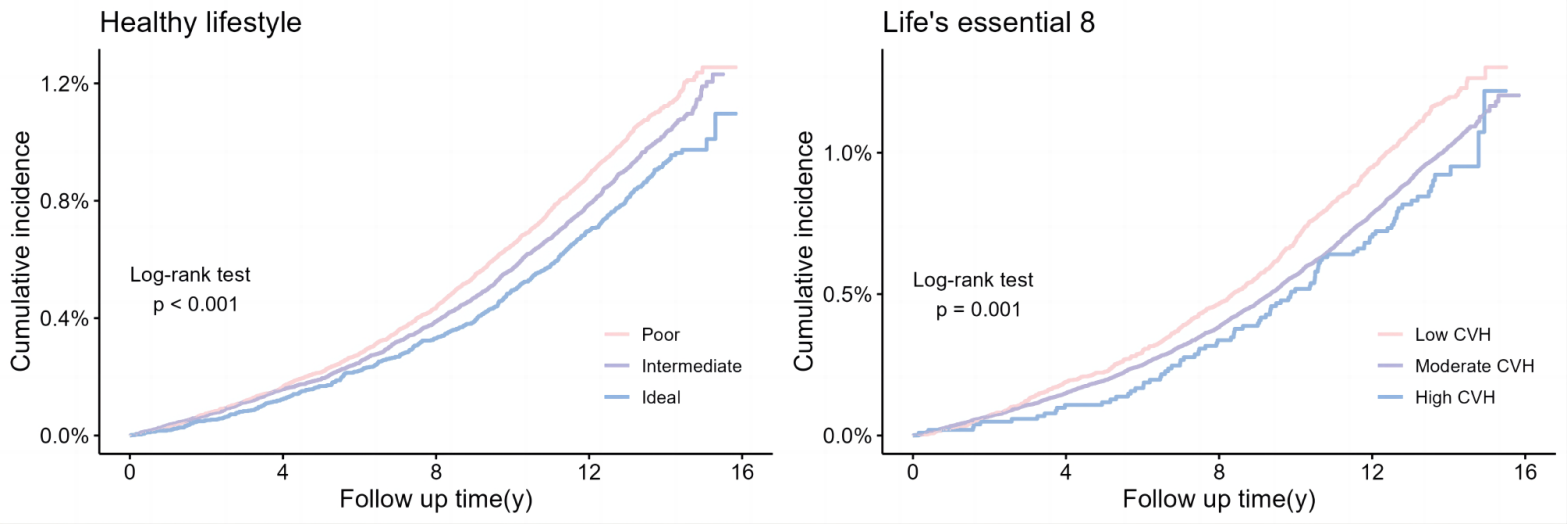
Cumulative incidence of migraine according to healthy lifestyle categories and life’s essential 8 (LE8) categories. * Analyses were stratified by age (> 55 and < = 55) and adjusted for sex, ethnicity, Townsend deprivation index, education, income, cardiovascular disease, cancer, other serious disease

**Fig. 3:**
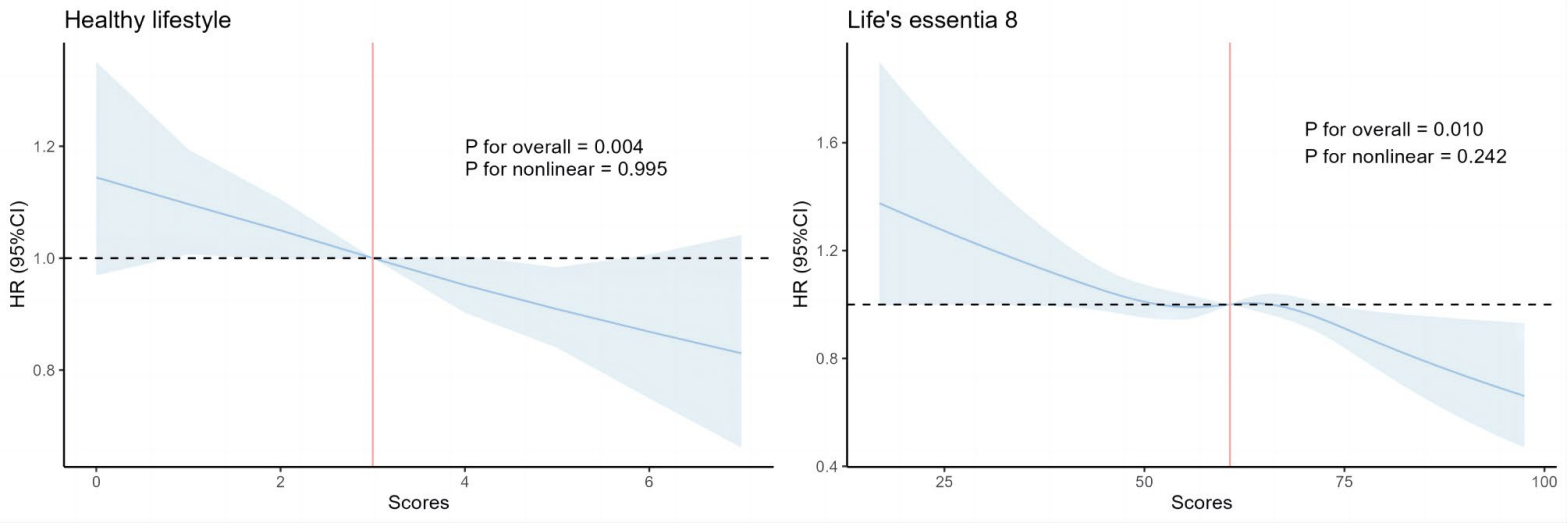
Restricted cubic splines of healthy lifestyle scores and life’s essential 8 scores with migraine. * Analyses were stratified by age (> 55 and < = 55) and adjusted for sex, ethnicity, Townsend deprivation index, education, income, cardiovascular disease, cancer, other serious disease

When the models were stratified based on age and adjusted for sex and ethnicity, the hazard ratios (HRs) for the poor, intermediate, and ideal Healthy lifestyle were 1 (reference), 0.85 (95% CI: 0.78,0.91), and 0.71 (95% CI: 0.64,0.79) respectively. The ideal Healthy lifestyle category exhibited a significant association with a reduced risk of migraine. After further accounting for the Townsend deprivation index, education, and income, this association weakened but still had statistical significance. The HRs for the three categories were 1 (reference), 0.88 (95% CI: 0.81,0.95), and 0.75 (95% CI: 0.68,0.84). When taking into account baseline cardiovascular diseases, cancer, and other severe diseases, the risk of migraine decreased by 9% for individuals in the intermediate category (HR: 0.91; 95% CI: 0.85,0.99) and by 19% for those in the ideal category (HR: 0.81; 95% CI: 0.73,0.91) compared to those in the poor category. If all participants were to adopt an ideal Healthy lifestyle, it is estimated that approximately 11.38% of migraine cases could be prevented (Table 2). Throughout the duration of the study, it was observed that individuals classified under the poor category showed the highest cumulative incidence rate (Fig. 2). Furthermore, when considering health lifestyle scores as a continuous variable, a rise in the scores was linked to a decline in the risk of experiencing migraine (Table 2; Fig. 3).

**Table 2:** Association between healthy lifestyle categories, life’s essential 8 categories with the risk of migraine

	Events (%)	Model 1		Model 2		Model 3		PAF% (95%CI)
		HR (95%CI)	*P* value	HR (95%CI)	*P* value	HR (95%CI)	*P* value	
**Healthy lifestyle categories**								11.38 (3.94,18.81)
Poor	1237 (38.4)	1(reference)		1(reference)		1(reference)		
Intermediate	1509 (46.8)	0.85(0.78,0.91)	< 0.001***	0.88(0.81,0.95)	0.001**	0.91(0.85,0.99)	0.035*	
Ideal	479 (14.9)	0.71(0.64,0.79)	< 0.001***	0.75(0.68,0.84)	< 0.001***	0.81(0.73,0.91)	< 0.001***	
*P* for trend		< 0.001***		< 0.001***		< 0.001***		
Per 1-point increase	3225	0.92(0.90,0.94)	< 0.001***	0.94(0.91,0.96)	< 0.001***	0.95(0.93,0.98)	< 0.001***	
**LE8 categories**								22.05 (6.01,38.09)
Low CVH	599 (18.6)	1(reference)		1(reference)		1(reference)		
Moderate CVH	2537 (78.7)	0.83(0.76,0.91)	< 0.001***	0.86(0.79,0.94)	0.001**	0.93(0.85,1.02)	0.144	
High CVH	89 (2.8)	0.65(0.52,0.82)	< 0.001***	0.65(0.52,0.82)	< 0.001***	0.73(0.58,0.92)	0.011*	
*P* for trend		< 0.001***		< 0.001***		0.006**		
Per 1-point increase	3225	0.99(0.99,0.99)	< 0.001***	0.99(0.99,0.99)	< 0.001***	1.00(0.99,1.00)	0.004**	

*Abbreviation* HR, hazard ratio; CI, confidence interval; CVH, cardiovascular health; PAF, population attributable fraction

All models were stratified by age (> 55 and < = 55)

Model 1: adjusted for sex, ethnicity;

Model 2: adjusted for sex, ethnicity, Townsend deprivation index, education, income;

Model 3: adjusted for sex, ethnicity, Townsend deprivation index, education, income, cardiovascular disease, cancer, other serious disease

Bold values indicate significance, with a P value < 0.05. *P < 0.05, **P < 0.01, ***P < 0.001

### Life’s essential 8 scores and migraine

The Life’s Essential 8 (LE8) scores categorized individuals into three groups: low, moderate, and high CVH, with 599, 2537, and 89 cases of reported migraine. Consistent with Healthy lifestyle, participants with high CVH demonstrated a significantly lower incidence of migraine (Fig. 2). The HRs for the low, moderate, and high categories were 1 (reference), 0.83 (95% CI: 0.76,0.91), and 0.65 (95% CI: 0.52,0.82) for model 1. Similarly, in model 2, the HRs for the same categories were 1 (reference), 0.86 (95% CI: 0.79,0.94), and 0.65 (95% CI: 0.52,0.82) for model 2, respectively. For model 3, the HRs were 1 (reference), 0.93 (95% CI: 0.85,1.02), and 0.73 (95% CI: 0.58,0.92). Furthermore, if all participants had high CVH, the risk of migraine would decrease by 22.05% (Table 2). The restricted cubic spline plots clearly demonstrate a negative correlation between increasing LE8 scores and migraine risk (Fig. 3).

### Individual lifestyle factors and migraine

Compared to participants at ideal levels, participants who are overweight (HR, 1.13; 95% CI: 1.03,1.24; PAF: 5.65%), those with poor physical activity (HR,1.13; 95% CI:1.02,1.25; PAF: 0.81%), those with excessively long or short sleep duration (long: HR, 1.27; 95% CI: 1.12,1.44; short: HR, 1.38; 95% CI: 1.28,1.49; PAF: 10.16%), those with low sleep scores (HR, 1.62; 95% CI: 1.39,1.89; PAF: 16.39%), and those with prolonged sedentary time (HR, 1.16; 95% CI: 1.08,1.25; PAF: 6.57%) have an increased risk of migraine. Conversely, alcohol consumption, diet scores, and blood pressure demonstrate an inverse relationship, with higher alcohol consumption (HR, 0.74; 95% CI: 0.68,0.81; PAF: -3.43%), lower diet score (HR, 0.89; 95% CI: 0.82,0.98; PAF: -10.42%), and higher blood pressure (HR, 0.86; 95% CI: 0.79,0.95; PAF: -12.12%) are linked to a reduced risk of migraine (Table 3). Similarly, in the restricted cubic sample plot, we can observe consistent findings. In addition, we discovered a correlation in the shape of a U between sleep duration and the risk of experiencing migraine (FigureS2).

**Table 3:** Association between individual lifestyle factors and migraine

Category	Model 1		Model 2		Model 3		PAF(95%CI)
	HR (95% CI)	*P* value	HR (95% CI)	*P* value	HR (95% CI)	*P* value	
Body mass index							5.65(0.84,10.46)
<25.0 kg/m2	1(reference)		1(reference)		1(reference)		
25.0–29.9 kg/m^2^	1.11(1.02,1.21)	0.015*	1.10(1.01,1.19)	0.039*	1.06(0.98,1.15)	0.208	
≥ 30.0 kg/m^2^	1.31(1.20,1.44)	< 0.001***	1.24(1.13,1.36)	< 0.001***	1.13(1.03,1.24)	0.016*	
Alcohol consumption*							-3.43(-6.40,-0.46)
Ideal	1(reference)		1(reference)		1(reference)		
Never	1.53(1.39,1.68)	< 0.001***	1.42(1.29,1.56)	< 0.001***	1.36(1.24,1.50)	< 0.001***	
Excessive	0.72(0.66,0.78)	< 0.001***	0.73(0.67,0.80)	< 0.001***	0.74(0.68,0.81)	< 0.001***	
Smoking status							-0.05(-3.21,3.10)
Never smoker	1(reference)		1(reference)		1(reference)		
Former smoker	1.07(1.00,1.16)	0.061	1.04(0.97,1.13)	0.411	1.02(0.95,1.10)	0.693	
Current smoker	1.13(1.01,1.27)	0.053	1.00(0.89,1.13)	0.999	0.99(0.88,1.12)	0.929	
Physical activity*							0.81(-2.17,3.80)
Ideal	1(reference)		1(reference)		1(reference)		
Intermediate	0.99(0.92,1.07)	0.793	1.00(0.93,1.08)	0.973	0.98(0.91,1.06)	0.786	
Poor	1.21(1.09,1.34)	0.001**	1.19(1.08,1.33)	0.002**	1.13(1.02,1.25)	0.043*	
Healthy diet scores							-10.42(-18.89,-1.95)
5–10 components	1(reference)		1(reference)		1(reference)		
0–4 components	0.88(0.81,0.97)	0.014*	0.89(0.81,0.98)	0.024*	0.89(0.82,0.98)	0.031*	
Sleep duration							10.16(7.63,12.70)
7–8 h/day	1(reference)		1(reference)		1(reference)		
≤ 6 h/day	1.48(1.37,1.59)	< 0.001***	1.42(1.32,1.53)	< 0.001***	1.38(1.28,1.49)	< 0.001***	
> 8 h/day	1.41(1.25,1.60)	< 0.001***	1.34(1.18,1.52)	< 0.001***	1.27(1.12,1.44)	< 0.001***	
Sleep scores							16.39(12.05,20.74)
4–5 points	1(reference)		1(reference)		1(reference)		
2–3 points	1.37(1.27,1.48)	< 0.001***	1.34(1.24,1.44)	< 0.001***	1.29(1.20,1.40)	< 0.001***	
0–1 points	1.91(1.64,2.23)	< 0.001***	1.78(1.53,2.08)	< 0.001***	1.62(1.39,1.89)	< 0.001***	
Sedentary time							6.57(3.12,10.02)
< 4 h/day	1(reference)		1(reference)		1(reference)		
≥ 4 h/day	1.26(1.18,1.35)	< 0.001***	1.20(1.12,1.29)	< 0.001***	1.16(1.08,1.25)	< 0.001***	
Non–HDL cholesterol							4.80(-1.62,11.22)
< 130 mg/dL	1(reference)		1(reference)		1(reference)		
130-189 mg/dL	0.99(0.90,1.08)	0.904	1.00(0.92,1.10)	0.969	1.04(0.96,1.14)	0.433	
≥ 190 mg/dL	0.99(0.90,1.11)	0.904	1.00(0.90,1.11)	0.969	1.06(0.95,1.14)	0.428	
HbA1c							-1.58(-3.28,0.12)
< 5.7%	1(reference)		1(reference)		1(reference)		
5.7–6.4%	1.00(0.90,1.11)	0.956	0.96(0.87,1.07)	0.648	0.92(0.83,1.02)	0.161	
≥ 6.5%	1.26(1.06,1.50)	0.018*	1.17(0.98,1.39)	0.132	1.03(0.87,1.23)	0.822	
Blood pressure							-12.12(-20.54,-3.70)
SBP < 120/DBP < 80mmHg	1(reference)		1(reference)		1(reference)		
Other	0.91(0.83,0.99)	0.037*	0.90(0.82,0.98)	0.032*	0.86(0.79,0.95)	0.003**	

*Abbreviation* HR, hazard ratio; CI, confidence interval; PAF, population attributable fraction; SBP, systolic blood pressure; DBP, diastolic blood pressure

*Alcohol consumption: Ideal:0 < women ≤ 14 g/day; 0 < men ≤ 28 g/day; Poor: Women>14 g/day; Men>28 g/day

*Physical activity: Ideal: ≥150 min of moderate physical activity; or ≥ 75 min of vigorous physical activity; or ≥ 150 min of combined moderate and vigorous physical activity; Intermediate: 0 < mins of moderate physical activity < 150; or 0 < mins of moderate < 75; or 0 < mins of combined moderate and vigorous physical activity < 150;Poor: No report of moderate or vigorous physical activity

All models were stratified by age (> 55 and < = 55)

Model 1: adjusted for sex, ethnicity;

Model 2: adjusted for sex, ethnicity, Townsend deprivation index, education, income;

Model 3: adjusted for sex, ethnicity, Townsend deprivation index, education, income, cardiovascular disease, cancer, other serious disease

Bold values indicate significance, with a P value <0.05. *P < 0.05, **P < 0.01, ***P < 0.001

### Subgroup analyses

**Fig. 4:**
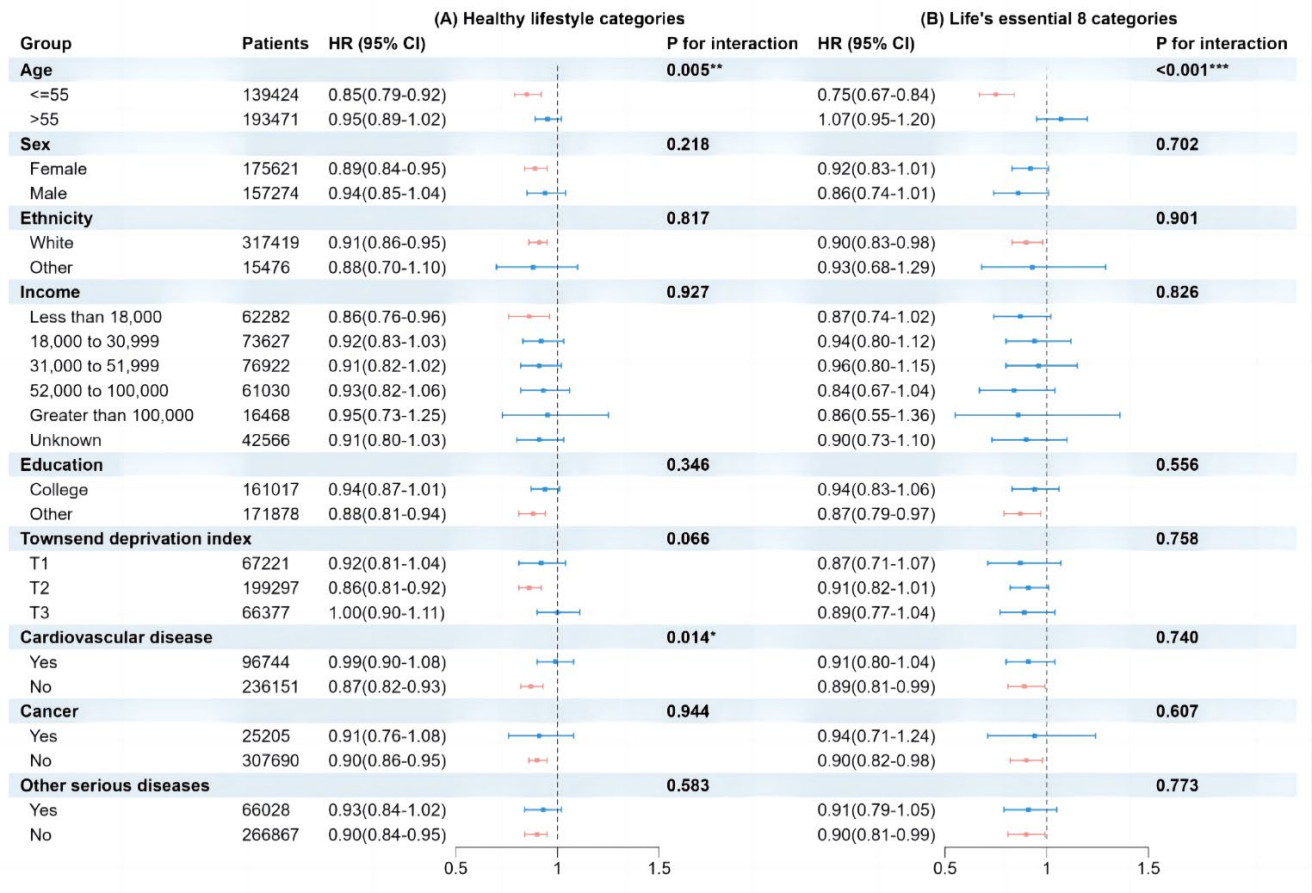
Stratified analyses of healthy lifestyle categories (per category increment) (**A**) and life’s essential 8 categories (per category increment) (**B**) with migraine. * Adjusted for age, sex, ethnicity, Townsend deprivation index, education, income, cardiovascular disease, cancer, other serious disease, if not already stratified. *P < 0.05, **P < 0.01, ***P < 0.001

To evaluate the effect of Healthy lifestyle categories and LE8 categories on migraine, we performed stratified and interaction analyses, taking into account various covariates. These covariates include age, sex, ethnicity, Townsend deprivation index, education, average household income, baseline cardiovascular disease, cancer, and other serious diseases. Our study findings suggest that the association between Healthy lifestyle categories and LE8 categories with migraine is notably stronger in participants aged 55 years or younger, compared to those aged over 55 years (interaction *p* = 0.005 and *p* < 0.001). Nevertheless, we we found no significant connections between other covariates and the risk of experiencing migraine, regardless of whether they fell under the Healthy lifestyle categories or LE8 categories (Fig. 4, Table S7).

### Sensitivity analyses

In order to evaluate the strength of these associations, we conducted several sensitivity analyses. Firstly, we utilized the chained equation algorithm to impute missing data for all exposure variables and covariates (Table S8). Secondly, we excluded individuals who reported migraine within two years (Table S9). Lastly, participants with other headaches at baseline were also excluded (Table S10). Through these sensitivity analyses, we determined that these exclusions had no significant impact on our study findings.

## Discussion

In this prospective cohort study, we excluded participants with pre-existing migraine and examined the connection between scores in Healthy lifestyle and LE8 with the risk of migraine. Specifically, adhering to a higher category of Healthy lifestyle and LE8 have been found to significantly decrease the risk of migraine by approximately 11.38% and 22.05%, respectively. When examining each lifestyle factor independently, maintaining an ideal BMI, physical activity, sleep duration, sleep pattern, and sedentary time have all been associated with a substantial reduction in the risk of migraine, with reductions of 5.65%, 0.81%, 10.16%, 16.39%, and 6.57%. Conversely, engaging in ideal alcohol consumption, diet, and blood pressure have been associated with a higher risk of experiencing migraine, with corresponding increases of 3.43%, 10.42%, and 12.12%.

Currently, there is a shortage of prospective research investigating the association between total lifestyle and migraine. Existing research mainly concentrates on assessing the effects of specific lifestyle changes on migraine, with fewer studies comprehensively considering multiple lifestyle factors. However, some studies suggest that factors such as BMI, sleep, diet, physical activity and stress are associated with migraine [10, 25–27]. Furthermore, the “Sleep, Exercise, Eat, Diary and Stress (SEEDS)” lifestyle modification approach posits that the monitoring, adaptation, and enhancement of sleep, diet, physical activity, and stress patterns can effectively mitigate the impact of migraine [27]. By conducting an extensive analysis of a substantial prospective cohort study and thoroughly considering various individual lifestyle factors, Healthy lifestyle scores, and LE8 scores, this research yields more precise and dependable findings to inform the management of migraine through lifestyle adjustments.

Sleep patterns have garnered significant attention, with insufficient sleep, poor sleep quality, and sleep disorders being associated with increased frequency and intensity of migraine [7]. We evaluated the healthy sleep patterns based on five factors: sleep duration, chronotype preference, insomnia symptoms, information on snoring, daytime sleepiness. Our findings suggest that both excessively long or short sleep durations, as well as unhealthy sleep patterns, can increase the risk of migraine. Although some studies indicate that short sleep duration predicts migraine occurrence [28], excessive sleep can also trigger migraine [29], yet the specific impact of sleep duration on migraine remains unclear. Chronotype refers to an individual’s intrinsic circadian rhythm and how it synchronizes with the 24-hour day. Some individuals are ‘night owls,’ staying up late into the night, while others are ‘morning larks,’ waking up early in the morning. Existing research has found that migraine sufferers are less likely to exhibit typical sleep chronotype compared to healthy controls, and their occurrence is associated with early chronotypes [30]. There may be a bidirectional relationship between migraine and insomnia. Compared to individuals without migraine but with insomnia, those without migraine but experiencing insomnia have a higher risk of migraine [31]. Habitual snoring is also considered a risk factor for chronic migraine [32]. The potential pathophysiological mechanisms that connect sleep disorders and migraine encompass crucial anatomical structures implicated in migraine pathogenesis and the regulation of the sleep-wake cycle, specifically the hypothalamus and brainstem regions. Additionally, at the molecular level, various substances, including orexin, melatonin, serotonin, dopamine, and adenosine, have been extensively investigated for their potential roles in mediating the relationship between sleep disorders and migraine [33].

Our investigation unveiled a noteworthy negative association between BMI and migraine, which is consistent with previous studies [34–36]. Obesity has been associated with migraine, potentially influenced by gender disparities and migraine frequency [34]. Additionally, our study established a link between poor physical activity, prolonged sitting and an increased risk of migraine. Prolonged sitting has been positively correlated with migraine risk, suggesting potential causal explanations. It can lead to poor posture, exacerbating tension in the neck and shoulder muscles, thereby increasing the frequency and severity of migraine attacks. Limiting daily sitting time to less than 6 h may help prevent around 22.1% of migraine occurrences [37]. The relationship between physical activity and migraine is complex. On one hand, physical activity can serve as a trigger for migraine, exacerbating acute episodes. Exercise may influence of the hypocretin pathway, increase lactate production, and elevate CGRP levels, thereby inducing migraine [9]. On the other hand, physical activity has a therapeutic effect on migraines. Regular physical exercise has been shown to be beneficial in reducing the occurrence of migraine attacks. Meta-analyses have demonstrated a decrease in average migraine days for patients engaging in aerobic exercise interventions [8, 38]. Moderate physical activity offers numerous advantages to individuals, encompassing cardiovascular well-being and enhanced sleep quality, all of which may indirectly contribute to the positive management of migraine.

The connection between diet and migraine is frequently observed, with alcohol and specific dietary patterns frequently identified as common triggers [39]. A comprehensive analysis revealed that approximately 21% of patients regarded alcohol as a trigger for migraine [40]. Ethanol has been implicated in triggering migraine through various mechanisms, including vasodilation, inhibition of antidiuretic hormone secretion, dehydration, and intracranial hypotension [41]. However, research findings concerning the association between alcohol consumption and migraine display inconsistency [42, 43]. The results of our study indicate a negative link between alcohol consumption and migraine, with the baseline alcohol consumption of the migraine group being comparatively lower than that of the non-migraine group. There has been a suggestion positing that the precise mechanism underlying this observation may imply that migraines prompt individuals to avoid alcohol, as opposed to alcohol exerting any protective influence against migraine [43].

Furthermore, specific dietary patterns characterized by healthfulness (ketogenic, low glycemic index, and DASH diets) and consistent eating habits may be linked to mitigating the impact of migraine [5, 44]. The mechanism through which diet influences migraine primarily involves avoiding trigger foods that may activate migraine attacks by affecting the plasma levels of key molecules involved in migraine pathogenesis, such as calcitonin gene-related peptide, nitric oxide (NO), and serotonin. Alternatively, diet may influence various aspects of brain homeostasis, including neuronal energy efficiency, excitability, inflammation, immune responses, and platelet aggregation [45]. Despite these potential benefits, our study revealed a positive relationship between a healthier dietary pattern and the risk of experiencing migraine. We relied on patients’ dietary recall at baseline to assess their adherence to a healthy dietary pattern, which may not have accurately captured individuals’ true dietary habits and intake levels. Furthermore, a healthy dietary pattern may inadvertently include foods associated with migraines, despite their generally recognized health benefits. Therefore, future research should employ more accurate and objective methods to assess participants’ dietary habits, thereby enhancing our understanding of the complex relationship between diet and migraine.

While it is commonly accepted that tobacco exposure can exacerbate headaches, the existing literature often presents conflicting findings. Nonetheless, a Mendelian randomization trial provides evidence supporting the detrimental impact of smoking on the development of migraine [46]. However, our study did not yield statistically significant differences in this regard.

While the connection between migraine and cardiovascular disorders is firmly established [47, 48], the relationship between blood pressure and migraine remains inconclusive. A notable inverse relationship between blood pressure and migraine at baseline has been documented in recent research [49]. However, other research findings are contradictory, with some studies indicating a positive association between high blood pressure and reduced migraine prevalence [50], while others only find an association with systolic blood pressure [51, 52]. Our study results reveal a negative association between blood pressure and the occurrence of headaches and migraine, suggesting complexity and heterogeneity in the relationship between blood pressure and migraine. One plausible explanation for our findings is a phenomenon known as hypertension-associated hyperalgesia, which posits that hypertensive patients exhibit a higher pain threshold compared to normotensive individuals. Hyperalgesia induced by baroreceptor activation secondary to elevated blood pressure may serve as a reward mechanism, potentially reinforced by recurrent stress [53]. Additionally, medications prescribed for hypertension management may confer a preventive effect on migraines. Among the classes of blood pressure-lowering medications, beta-blockers and angiotensin II receptor blockers are recommended by guidelines for migraine prophylaxis [54].

Several studies have indicated that individuals experiencing migraine exhibit heightened levels of total cholesterol, low-density lipoprotein cholesterol, or triglycerides, alongside reduced levels of high-density lipoprotein cholesterol [55, 56]. However, our research findings suggest that the concentration of non-HDL is not linked to the occurrence of migraines. This phenomenon could be attributed to the fact that non-HDL is derived by subtracting total cholesterol from high-density lipoprotein cholesterol, thereby implying that its relationship with migraine may be influenced by additional variables, such as distinct cholesterol types.

The existing body of research on the relationship between migraine and glucose-related characteristics lacks consensus, and the underlying mechanism is complex [57]. A cross-sectional study revealed no significant association between HbA1c% in individuals with diabetes and the occurrence of migraine [58]. In our study, the examination of HbA1c failed to demonstrate any significant association with migraine. However, it was observed that elevated levels of blood glucose exhibited a negative association with the incidence of migraine, implying that a moderate increase in blood glucose levels may confer a protective influence on migraine, potentially attributable to the heightened energy requirements associated with migraine.

The primary strengths of this study resides in its utilization of a large-scale prospective cohort study design, which thoroughly incorporates comprehensive Healthy lifestyle factors and the most up-to-date LE8 scoring system for evaluating migraine. Moreover, our study is advantageous due to an extended follow-up duration, a substantial sample size, and rigorous statistical power.

There exist certain limitations. Firstly, it is an observational study, thereby precluding the establishment of causation. Consequently, prudence is required when interpreting the findings to avert undue extrapolation. Secondly, with regard to lifestyle factors, the data gathered predominantly relies on questionnaire surveys encompassing alcohol consumption and dietary habits, thereby potentially introducing recall bias and classification errors. Thirdly, the determination of migraine relies on data obtained from self-reports and hospital inpatient records using the ICD-10 classification system, rather than the Third Edition of the International Classification of Headache Disorders (ICHD-3) criteria. It is important to acknowledge that this approach may lead to misdiagnosis as well as overlook individuals with migraine who have not been formally diagnosed. Consequently, our primary focus was on individuals who have received a diagnosis for migraines rather than those who remain undiagnosed. Fourthly, despite the comprehensive incorporation of various confounding factors in our analysis, the potential existence of unaccounted confounding factors remains. Finally, it’s worth noting that the population in the UK Biobank ranged from 37 to 73 years old and was predominantly Caucasian. While our study provides valuable insights into the influence of lifestyle factors on the risk of migraine onset later in life, its applicability to all migraineurs is uncertain. Therefore, it is imperative to validate our reported findings through subsequent investigations.

## Conclusion

In conclusion, poor Healthy lifestyle and Life’s Essential 8 are associated with higher risk of new-onset migraine. Consequently, it is imperative to promote the adoption of ideal Healthy lifestyle and the enhancement of LE8 scores among individuals, as these measures hold potential for mitigating the onset of migraine.

### Electronic supplementary material

Below is the link to the electronic supplementary material.

Supplementary Material 1

## Data Availability

All data is in the UK Biobank database, and researchers can apply to use UK Biobank resources and access the data used at https://www.ukbiobank.ac. No other data is available.

## Abbreviations

LE8: Life’s essential 8
AHA: American Heart Association
CVH: Cardiovascular Health
PAF: Population attributable fraction
BMI: Body mass index
HDL: High-density lipoprotein
HbA1c: Glycated hemoglobin
ICD10: Tenth Revision of the International Classification of Diseases
ICHD-3: Third Edition of the International Classification of Headache Disorders
VIF: Variance inflation factor
SD: Standard deviation
IQR: Interquartile range
HR: Hazard ratio
CI: Confidence interval
