# Supplementary material for: Poor healthy lifestyle and life’s essential 8 are associated with higher risk of new-onset migraine: a prospective cohort study
Source: J Headache Pain. 2024 May 17;25(1):82. doi: 10.1186/s10194-024-01785-4 (PMC11100122; doi:10.1186/s10194-024-01785-4)
Supplement: Supplementary file 1 — Supplementary Material 1 [file 10194_2024_1785_MOESM1_ESM.docx]

**Supplementary Materials: Poor healthy lifestyle and Life's Essential 8 are Associated with Higher Risk of new-onset Migraine: A prospective study**

[Supplemental table 1. Definitions of components used in the UK Biobank study of Healthy lifestyle scores 2](#_Toc2281)

[Supplemental table 2. Components of modified dietary scores used in the UK Biobank study 3](#_Toc32043)

[Supplemental table 3. Healthy sleep pattern definitions used in the UK Biobank study 5](#_Toc13888)

[Supplemental table 4. Quantitative Assessment of Life’s Essential 8 (LE8) scores 6](#_Toc6630)

[Supplemental table 5. Covariates multicollinearity test results in Model 2. 9](#_Toc4344)

[Supplemental table 6. Covariates multicollinearity test results in Model 3. 10](#_Toc6564)

[Supplementary Table 7. Associations between Healthy lifestyle categories (per category increment), Life’s essential 8 categories (per category increment) and Migraine by subgroups. 11](#_Toc31272)

[Supplemental Figure 1. Distribution of Healthy lifestyle scores, LE8 scores and individual lifestyle factors among UK Biobank participants at baseline. BMI = Body mass index. 13](#_Toc23673)

[Supplemental figure 2. Association between individual lifestyle factors and migraine in Cox models with restricted cubic splines. 14](#_Toc3996)

[Supplemental Figure 3. Population attributable fractions for individual lifestyle factors with migraine. 15](#_Toc30430)

[Supplementary Table 8. Association between healthy lifestyle categories ( poor, intermediate, ideal), Life’s essential 8 (LE8) categories (low, moderate, and high CVH) with the risk of migraine. 16](#_Toc21064)

[Supplementary Table 9. Association between healthy lifestyle categories ( poor, intermediate, ideal), Life’s essential 8 (LE8) categories (low, moderate, and high CVH) with the risk of migraine. (2-year landmark analysis) 17](#_Toc4844)

[Supplementary Table 10. Association between healthy lifestyle categories ( poor, intermediate, ideal), Life’s essential 8 (LE8) categories (low, moderate, and high CVH) with the risk of migraine. (excluded other headaches) 18](#_Toc1287)

# Supplemental table 1. Definitions of components used in the UK Biobank study of Healthy lifestyle scores

| Lifestyle factors | Classification of healthy lifestyle score | | UK Biobank field code |
| --- | --- | --- | --- |
|  | Poor | Ideal |  |
| BMI(Body Mass Index) | ≥ 25.0 kg/m^2^ | < 25.0 kg/m^2^ | 21001 |
| Cigarette smoking | Current/former smoker | never smoker | 20116 |
| Alcohol consumption | Women＞14g/day;  Men＞28g/day; Never | 0<women≤14g/day; 0<men≤28g/day | 20117,1558,1568,1578,1588  1598,1608,4407,4418,4429,4440,4451 |
| Physical activity | No report of moderate or vigorous physical activity;  0 < mins of moderate physical activity < 150; or  0 < mins of moderate < 75; or  0 < mins of combined moderate and vigorous physical activity < 150 | ≥150 mins of moderate physical activity; or  ≥75 mins of vigorous physical activity; or  ≥150 mins of combined moderate and vigorous physical activity | 884, 894, 904, 914 |
| Healthy diet score | 0-4 components | 5-10 components | 1309,1319,1289,1299,1329,1339,1349,1369,1379,1389,1438,1448,1458,1468 |
| Sleep pattern score | 0-3 points | 4-5 points | 1160,1180,1200,1210,1220 |
| Sedentary time | ≥ 4 h/day | < 4 h/day | 1080.1090 |

# Supplemental table 2. Components of modified dietary scores used in the UK Biobank study

| **Diet component** | **Intake goal** | **Field IDs** | **Amount per serving** |
| --- | --- | --- | --- |
| Fruit | 3 servings/day | 1309 (pieces fresh fruit/day)  1319 (pieces dried fruit/day) | 1309 – 1 piece  1319 – 5 pieces |
| Vegetable | 3 servings/day | 1289 (tablespoons cooked vegetables/day)  1299 (salad/raw vegetables/day) | 3 heaped tablespoons |
| Whole grains | 3 servings/day | 1438, 1448 (whole meal/wholegrain bread slices/week)  1458, 1468 (bran/oat/muesli cereal bowls/week) | 1438/1448 – 1 slice/day  1458/1468 – 1 bowl/day |
| (Shell)fish | ≥2 servings/week | 1329 (oily fish/week)  1339 (non-oily fish/week) | Once/week |
| Dairy | 2 servings/day | 1408 (cheese/week)  1418 (milk type) | 1408 – 1 piece/day  1418 – 1 glass/day if consumption of any type of milk |
| Vegetable oils | 2 servings/day | 1428 (Flora Pro-Active/Benecol spread)  2654 (Flora Pro-Active/Benecol, soft margarine -, olive oil based -, polyunsaturated/sunflower oil based -, other low/reduced fat spread)  1438 (bread slices/week) | 1 serving/day if in combination with eating at least 2 slices of bread (ID 1438) |
| Refined grains | ≤2 servings/day | 1438, 1448 (white, brown, other bread slices/week)  1458, 1468 (biscuit, other cereals/week) | 1438/1448 – 1 slice/day  1458/1468 – 1 bowl/day |
| Processed meats | ≤1 serving/week | 1349 (processed meat/week or daily)  3680 (age when last ate meat) | 1349 – 1 piece/day  3680 – 0 pieces/day if indicated having never eaten meat |
| Unprocessed meats | ≤2 servings/day | 1359 (poultry/week or day)  1369 (beef/week or day)  1379 (lamb or mutton/week or day)  1389 (pork/week or day)  3680 (age when last ate meat) | 1359-1389 – once/week  3680 – 0 pieces/day if indicated having never eaten meat |

Field IDs and serving sizes used per diet component in UK Biobank with available data from the general baseline questionnaire. If participants achieved the intake goal, they were considered to have an adequate intake of the diet component.

Scoring criteria for dietary recommendations for cardiovascular health: 1: If intake goal met; 0: If intake goal not met. (Range: 0-10)

# Supplemental table 3. Healthy sleep pattern definitions used in the UK Biobank study

| Sleep behaviours | High-risk sleep factors^a^ | Low-risk sleep factors | Field IDs |
| --- | --- | --- | --- |
| Chronotype preference | more an “evening” than “morning” person  definitely an “evening” person | definitely a “morning” person  more a “morning” than “evening” person | 1180 |
| Sleep duration | short (<7h/day)  long (>=9h/day) | normal (7–8h/day) | 1160 |
| Insomnia symptoms | Sometimes  usually | Never/rarely | 1200 |
| Information on snoring | yes | no | 1210 |
| daytime sleepiness | Often  all of the time | never/rarely  sometimes | 1220 |

^a^For each sleep factor, and the participant received a score of 1 if he or she was classified as low risk for that factor or 0 if at high risk for that factor.

# Supplemental table 4. Quantitative Assessment of Life’s Essential 8 (LE8) scores

| LE8 metric | Method of measurement | Quantification of LE8 metric | Score and classification |
| --- | --- | --- | --- |
| Diet | Measurement: A more recent definition of ideal intake of dietary components for cardiovascular health | Scoring:  Points diets score (points)  100 8–10  80 6–7  50 4–5  25 2–3  0 0–1 | The LE8 score is scaled from 0 to 100 points, calculated as the unweighted average of all 8 component metric scores.  In the present study, overall LE8 scores of 80 to 100 are considered High CVH; 60 to 79, Moderate CVH; and 0 to 59 points, low CVH. |
| Physical activity (PA) | Self-reported minutes of moderate or vigorous PA per week | Scoring:  Points Minutes  100 ≥150  90 120–149  80 90–119  60 60–89  40 30–59  20 1–29  0 0 |  |
| Tobacco/nicotine exposure | Self-reported use of cigarettes; or secondhand smoke exposure | Scoring:  Points Status  100 Never smoker  75 Former smoker, quit ≥5 y  50 Former smoker, quit 1–<5 y  25 Former smoker, quit <1 y  0 Current smoker  Subtract 20 points (unless score is 0) for  living with active indoor smoker in home |  |
| Sleep health | Self-reported average hours of sleep per night | Scoring:  Points Level  100 7–<9  90 9–<10  70 6–<7  40 5–<6 or ≥10  20 4–<5  0 <4 |  |
| Body mass index | Measurement: Body weight (kilograms) divided by height squared (meters squared) | Scoring:  Points Level  100 <25  70 25.0–29.9  30 30.0–34.9  15 35.0–39.9  0 ≥40.0 |  |
| Blood lipids (non-HDL cholesterol) | Measurement: Plasma total and HDL cholesterol with calculation of non–HDL cholesterol | Metric: Non–HDL cholesterol (mg/dL)  Scoring:  Points Level  100 <130  60 130–159  40 160–189  20 190–219  0 ≥220  If drug-treated level, subtract 20 points |  |
| Blood glucose | Measurement: HbA1c and history of Diabetes | Metric: HbA1c (%)  Scoring:  Points Level  100 No history of diabetes HbA1c <5.7  60 No diabetes and HbA1c 5.7–6.4 (prediabetes)  40 Diabetes with HbA1c <7.0  30 Diabetes with HbA1c 7.0–7.9  20 Diabetes with HbA1c 8.0–8.9  10 Diabetes with Hb A1c 9.0–9.9  0 Diabetes with HbA1c ≥10.0 |  |
| Blood pressure (BP) | Measurement: Appropriately measured systolic and diastolic blood pressures | Metric: Systolic and diastolic BPs (mm Hg)  Scoring:  Points Level  100 <120/<80 (optimal)  75 120–129/<80 (elevated)  50 130–139 or 80–89 (stage 1 hypertension)  25 140–159 or 90–99  0 ≥160 or ≥100  Subtract 20 points if treated level |  |

# Supplemental table 5. Covariates multicollinearity test results in Model 2.

| VIF | Sex | Ethnicity | Townsend deprivation index | Income | Education |
| --- | --- | --- | --- | --- | --- |
| **Healthy lifestyle** |  |  |  |  |  |
| Healthy lifestyle scores | 1.0134 | 1.0207 | 1.0574 | 1.0188 | 1.0587 |
| Healthy lifestyle categories | 1.0123 | 1.0210 | 1.0565 | 1.0187 | 1.0580 |
| **Life’s essential 8** |  |  |  |  |  |
| LE8 scores | 1.0098 | 1.0227 | 1.0530 | 1.0188 | 1.0559 |
| LE8 categories | 1.0083 | 1.0220 | 1.0540 | 1.0186 | 1.0545 |
| **Individual lifestyle factors** |  |  |  |  |  |
| Body mass index | 1.0168 | 1.0212 | 1.0558 | 1.0188 | 1.0563 |
| Alcohol consumption | 1.0105 | 1.0373 | 1.0564 | 1.0211 | 1.0540 |
| Smoking status | 1.0109 | 1.0247 | 1.0638 | 1.0189 | 1.0549 |
| Physical activity | 1.0070 | 1.0207 | 1.0526 | 1.0186 | 1.0544 |
| Healthy diet scores | 1.0084 | 1.0209 | 1.0524 | 1.0184 | 1.0547 |
| Sleep duration | 1.0069 | 1.0221 | 1.0549 | 1.0193 | 1.0545 |
| Sleep scores | 1.0082 | 1.0206 | 1.0539 | 1.0187 | 1.0538 |
| Sedentary time | 1.0104 | 1.0207 | 1.0531 | 1.0196 | 1.0575 |
| Non–HDL cholesterol | 1.0068 | 1.0214 | 1.0527 | 1.0185 | 1.0531 |
| HbA1c | 1.0090 | 1.0289 | 1.0537 | 1.0188 | 1.0534 |
| Blood pressure | 1.0187 | 1.0207 | 1.0525 | 1.0185 | 1.0541 |

VIF, variance inflation factor; LE8, Life’s essential 8.

# Supplemental table 6. Covariates multicollinearity test results in Model 3.

| VIF | Sex | Ethnicity | TDI | Income | Education | CVD | Cancer | OSD |
| --- | --- | --- | --- | --- | --- | --- | --- | --- |
| **Healthy lifestyle** |  |  |  |  |  |  |  |  |
| Healthy lifestyle scores | 1.0182 | 1.0216 | 1.0595 | 1.0198 | 1.0611 | 1.0243 | 1.0062 | 1.0169 |
| Healthy lifestyle categories | 1.0172 | 1.0218 | 1.0587 | 1.0197 | 1.0604 | 1.0230 | 1.0062 | 1.0165 |
| **Life’s essential 8** |  |  |  |  |  |  |  |  |
| LE8 scores | 1.0135 | 1.0232 | 1.0556 | 1.0197 | 1.0578 | 1.0485 | 1.0061 | 1.0157 |
| LE8 categories | 1.0133 | 1.0225 | 1.0565 | 1.0196 | 1.0566 | 1.0359 | 1.0061 | 1.0153 |
| **Individual lifestyle factors** |  |  |  |  |  |  |  |  |
| Body mass index | 1.0209 | 1.0219 | 1.0576 | 1.0197 | 1.0583 | 1.0418 | 1.0061 | 1.0159 |
| Alcohol consumption | 1.0161 | 1.0376 | 1.0588 | 1.0220 | 1.0565 | 1.0166 | 1.0061 | 1.0167 |
| Smoking status | 1.0170 | 1.0254 | 1.0667 | 1.0200 | 1.0576 | 1.0169 | 1.0063 | 1.0144 |
| Physical activity | 1.0129 | 1.0216 | 1.0553 | 1.0197 | 1.0569 | 1.0175 | 1.0061 | 1.0162 |
| Healthy diet scores | 1.0143 | 1.0218 | 1.0552 | 1.0195 | 1.0572 | 1.0162 | 1.0061 | 1.0140 |
| Sleep duration | 1.0127 | 1.0230 | 1.0575 | 1.0203 | 1.0570 | 1.0172 | 1.0062 | 1.0162 |
| Sleep scores | 1.0136 | 1.0215 | 1.0564 | 1.0197 | 1.0562 | 1.0186 | 1.0061 | 1.0171 |
| Sedentary time | 1.0156 | 1.0216 | 1.0558 | 1.0205 | 1.0599 | 1.0181 | 1.0061 | 1.0150 |
| Non–HDL cholesterol | 1.0125 | 1.0222 | 1.0553 | 1.0196 | 1.0556 | 1.0236 | 1.0061 | 1.0143 |
| HbA1c | 1.0139 | 1.0290 | 1.0560 | 1.0197 | 1.0558 | 1.0300 | 1.0062 | 1.0149 |
| Blood pressure | 1.0221 | 1.0215 | 1.0554 | 1.0196 | 1.0563 | 1.0303 | 1.0061 | 1.0141 |

VIF, variance inflation factor; TDI, Townsend deprivation index; CVD, cardiovascular diseases; OSD, other serious diseases; LE8, Life’s essential 8.

# Supplementary Table 7. Associations between Healthy lifestyle categories (per category increment), Life’s essential 8 categories (per category increment) and Migraine by subgroups.

| **Group** | **Patients** | **Healthy lifestyle categories** | | **LE8 categories** | |
| --- | --- | --- | --- | --- | --- |
|  |  | **HR(95%CI)** | **P for interaction** | **HR(95%CI)** | **P for interaction** |
| **Age** |  |  | **0.005**** |  | **<0.001***** |
| <=55 | 139424 | 0.85(0.79-0.92) |  | 0.75(0.67-0.84) |  |
| >55 | 193471 | 0.95(0.89-1.02) |  | 1.07(0.95-1.2) |  |
| **Sex** |  |  | 0.218 |  | 0.702 |
| Female | 175621 | 0.89(0.84-0.95) |  | 0.92(0.83-1.01) |  |
| Male | 157274 | 0.94(0.85-1.04) |  | 0.86(0.74-1.01) |  |
| **Ethnicity** |  |  | 0.817 |  | 0.901 |
| White | 317419 | 0.91(0.86-0.95) |  | 0.9(0.83-0.98) |  |
| Other | 15476 | 0.88(0.7-1.1) |  | 0.93(0.68-1.29) |  |
| **Income** |  |  | 0.927 |  | 0.826 |
| Less than 18,000 | 62282 | 0.86(0.76-0.96) |  | 0.87(0.74-1.02) |  |
| 18,000 to 30,999 | 73627 | 0.92(0.83-1.03) |  | 0.94(0.8-1.12) |  |
| 31,000 to 51,999 | 76922 | 0.91(0.82-1.02) |  | 0.96(0.8-1.15) |  |
| 52,000 to 100,000 | 61030 | 0.93(0.82-1.06) |  | 0.84(0.67-1.04) |  |
| Greater than 100,000 | 16468 | 0.95(0.73-1.25) |  | 0.86(0.55-1.36) |  |
| Unknown | 42566 | 0.91(0.8-1.03) |  | 0.9(0.73-1.1) |  |
| **Education** |  |  | 0.346 |  | 0.556 |
| College | 161017 | 0.94(0.87-1.01) |  | 0.94(0.83-1.06) |  |
| Other | 171878 | 0.88(0.81-0.94) |  | 0.87(0.79-0.97) |  |
| **Townsend deprivation index** |  |  | 0.066 |  | 0.758 |
| T1 | 67221 | 0.92(0.81-1.04) |  | 0.87(0.71-1.07) |  |
| T2 | 199297 | 0.86(0.81-0.92) |  | 0.91(0.82-1.01) |  |
| T3 | 66377 | 1(0.9-1.11) |  | 0.89(0.77-1.04) |  |
| **Cardiovascular disease** |  |  | **0.014*** |  | 0.740 |
| Yes | 96744 | 0.99(0.9-1.08) |  | 0.91(0.8-1.04) |  |
| No | 236151 | 0.87(0.82-0.93) |  | 0.89(0.81-0.99) |  |
| **Cancer** |  |  | 0.944 |  | 0.607 |
| Yes | 25205 | 0.91(0.76-1.08) |  | 0.94(0.71-1.24) |  |
| No | 307690 | 0.9(0.86-0.95) |  | 0.9(0.82-0.98) |  |
| **Other serious diseases** |  |  | 0.583 |  | 0.773 |
| Yes | 66028 | 0.93(0.84-1.02) |  | 0.91(0.79-1.05) |  |
| No | 266867 | 0.9(0.84-0.95) |  | 0.9(0.81-0.99) |  |

*Analyses were stratified by age, sex, ethnicity, Townsend deprivation index, education, income, cardiovascular disease, cancer, other serious diseases. *P<0.05, **P<0.01, ***P<0.001.


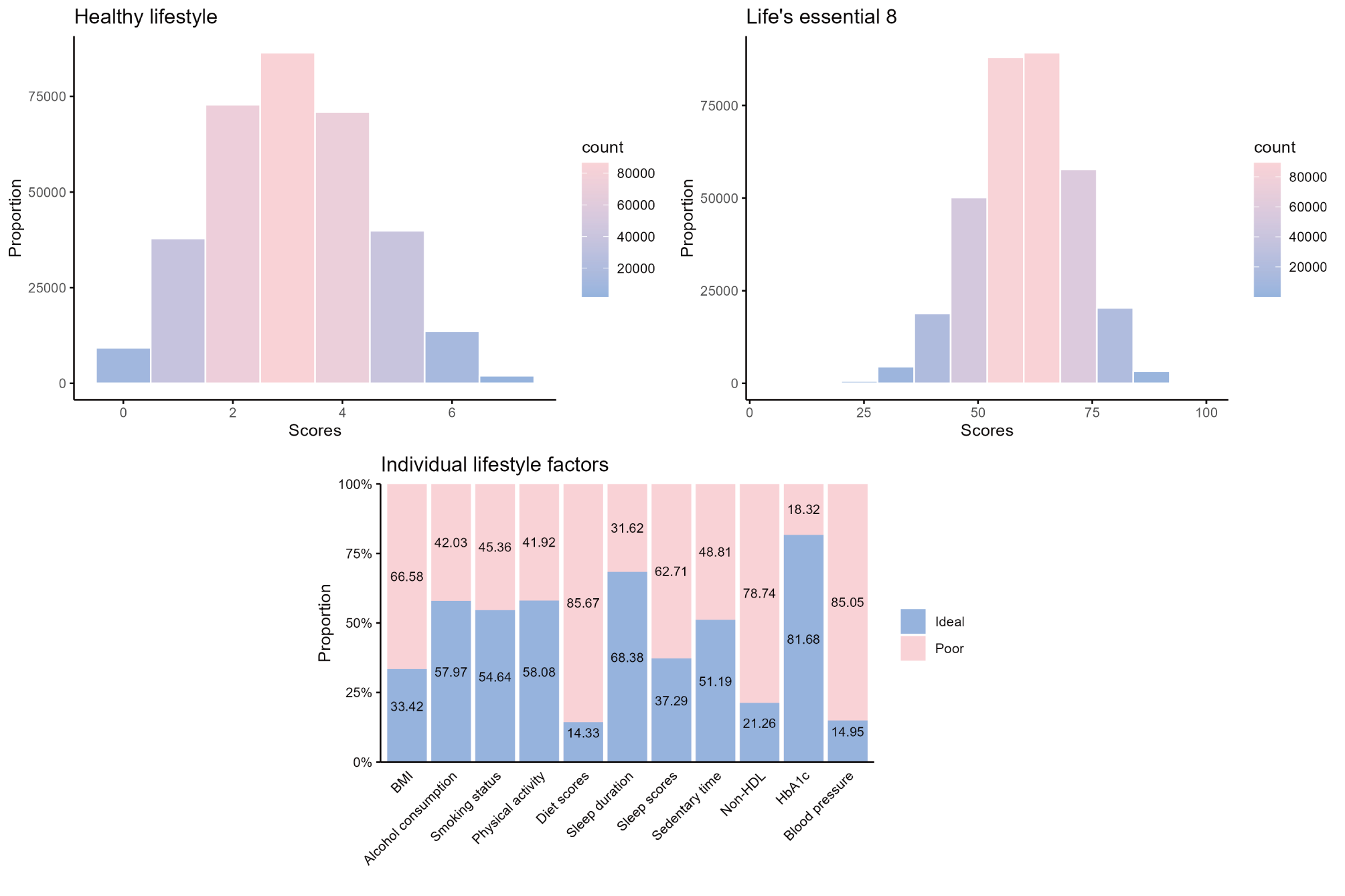


# Supplemental Figure 1. Distribution of Healthy lifestyle scores, LE8 scores and individual lifestyle factors among UK Biobank participants at baseline. BMI = Body mass index.

**
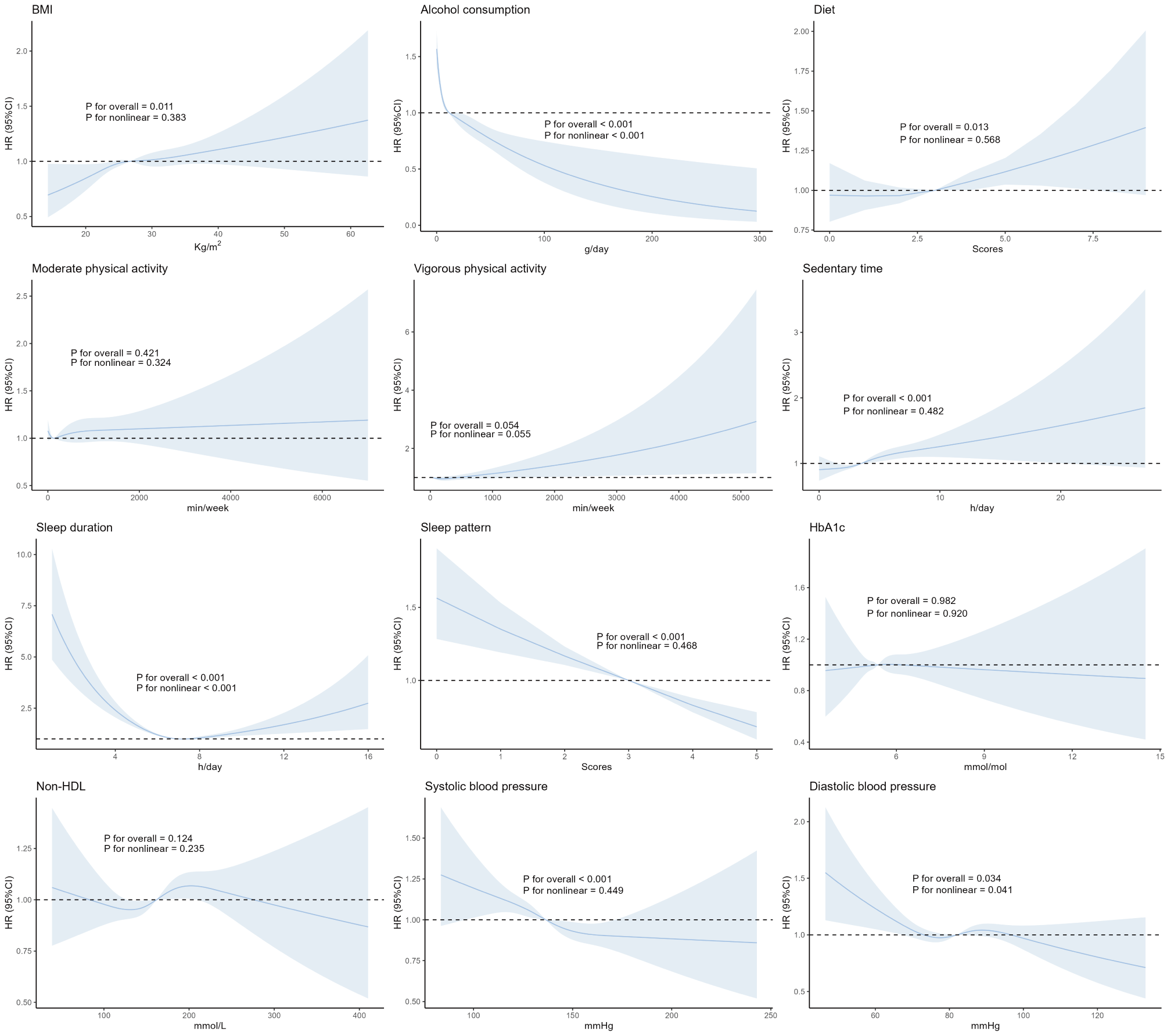
**

# Supplemental figure 2. Association between individual lifestyle factors and migraine in Cox models with restricted cubic splines.

*Analyses were stratified by age (>55 and <=55) and adjusted for sex, ethnicity, Townsend deprivation index, education, income, cardiovascular disease, cancer, other serious diseases.

**
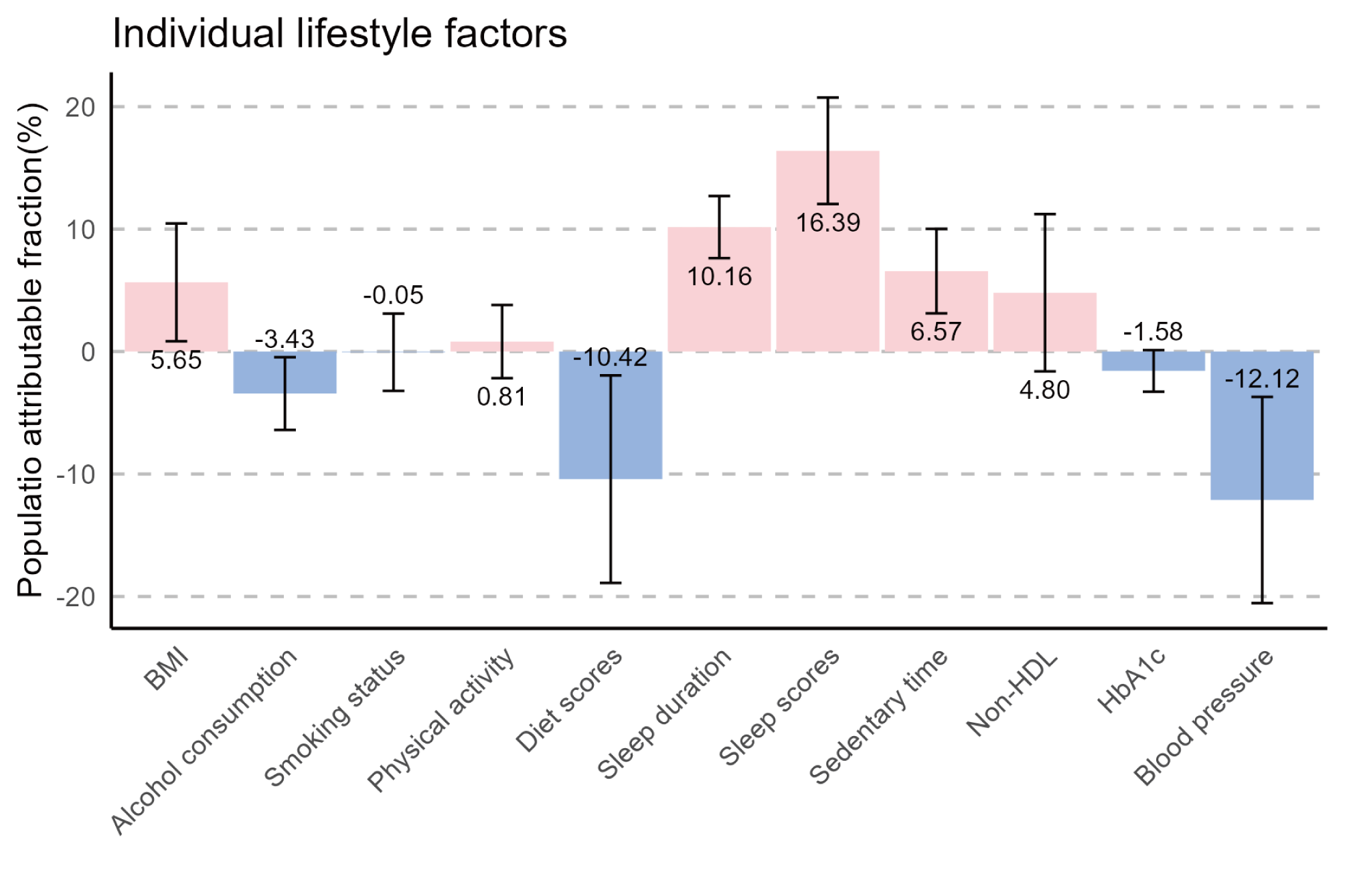
**

# Supplemental Figure 3. Population attributable fractions for individual lifestyle factors with migraine.

*Analyses were stratified by age (>55 and <=55) and adjusted for sex, ethnicity, Townsend deprivation index, education, income, cardiovascular disease, cancer, other serious diseases.

# Supplementary Table 8. Association between healthy lifestyle categories ( poor, intermediate, ideal), Life’s essential 8 (LE8) categories (low, moderate, and high CVH) with the risk of migraine.

|  | Events (%) | Model 1 |  | Model 2 |  | Model 3 |  |
| --- | --- | --- | --- | --- | --- | --- | --- |
|  |  | HR (95%CI) | *P* value | HR (95%CI) | *P* value | HR (95%CI) | *P* value |
| **Healthy lifestyle** | |  |  |  |  |  |  |
| Poor | 1392 (39.1) | 1(reference) |  | 1(reference) |  | 1(reference) |  |
| Intermediate | 1654 (46.5) | 0.84(0.78,0.90) | **<0.001***** | 0.88(0.81,0.94) | **<0.001***** | 0.91(0.85,0.98) | **0.018*** |
| Ideal | 511 (14.4) | 0.70(0.63,0.71) | **<0.001***** | 0.75(0.68,0.84) | **<0.001***** | 0.81(0.73,0.90) | **<0.001***** |
| *P* for trend |  | <0.001 |  | <0.001 |  | <0.001 |  |
| Per 1-point increase | 3557 | 0.91(0.,890.94) | **<0.001***** | 0.93(0.91,0.95) | **<0.001***** | 0.95(0.93,0.97) | **<0.001***** |
| **CVH categories** | |  |  |  |  |  |  |
| Low CVH | 681 (19.1) | 1(reference) |  | 1(reference) |  | 1(reference) |  |
| Moderate CVH | 2782 (78.2) | 0.84(0.77,0.91) | **<0.001***** | 0.87(0.80,0.94) | **0.002**** | 0.93(0.86,1.02) | 0.154 |
| High CVH | 94 (2.7) | 0.64(0.52,0.80) | **<0.001***** | 0.64(0.52,0.80) | **<0.001***** | 0.72(0.58,0.89) | **0.005**** |
| *P* for trend |  | <0.001 |  | <0.001 |  | 0.004 |  |
| Per 1-point increase | 3557 | 0.99(0.99,0.99) | **<0.001***** | 0.99(0.99,1.00) | **<0.001***** | 1.00(0.99,1.00) | **0.003**** |

Abbreviations: HR, hazard ratio; CI, confidence interval; CVH, cardiovascular health; LE8, Life’s Essential 8; PAF, population attributable fraction.

All models were stratified by age (>55 and <=55).

Model 1: adjusted for sex, ethnicity;

Model 2: adjusted for sex, ethnicity, Townsend deprivation index, education, income;

Model 3: adjusted for sex, ethnicity, Townsend deprivation index, education, income, cardiovascular disease, cancer, other serious disease.

*P<0.05, **P<0.01, ***P<0.001.

# Supplementary Table 9. Association between healthy lifestyle categories ( poor, intermediate, ideal), Life’s essential 8 (LE8) categories (low, moderate, and high CVH) with the risk of migraine. (2-year landmark analysis)

|  | Events (%) | Model 1 |  | Model 2 |  | Model 3 |  |
| --- | --- | --- | --- | --- | --- | --- | --- |
|  |  | HR (95%CI) | *P* value | HR (95%CI) | *P* value | HR (95%CI) | *P* value |
| **Healthy lifestyle** | |  |  |  |  |  |  |
| Poor | 1147 (38.2) | 1(reference) |  | 1(reference) |  | 1(reference) |  |
| Intermediate | 1402 (46.8) | 0.85(0.78,0.92) | **<0.001***** | 0.88(0.81,0.95) | **0.002**** | 0.91(0.84,0.99) | **0.042*** |
| Ideal | 450 (15.0) | 0.71(0.64,0.80) | **<0.001***** | 0.76(0.68,0.85) | **<0.001***** | 0.82(0.73,0.92) | **0.001**** |
| *P* for trend |  | <0.001 |  | <0.001 |  | 0.001 |  |
| Per 1-point increase | 2999 | 0.92(0.90,0.94) | **<0.001***** | 0.94(0.91,0.96) | **<0.001***** | 0.95(0.93,0.98) | **0.001**** |
| **CVH categories** | |  |  |  |  |  |  |
| Low CVH | 561 (18.7) | 1(reference) |  | 1(reference) |  | 1(reference) |  |
| Moderate CVH | 2354 (78.5) | 0.83(0.75,0.91) | **<0.001***** | 0.85(0.78,0.93) | **0.001**** | 0.92(0.83,1.01) | 0.109 |
| High CVH | 84 (2.8) | 0.66(0.52,0.83) | **0.001**** | 0.66(0.52,0.83) | **0.001**** | 0.74(0.58,0.93) | **0.018*** |
| *P* for trend |  | 0.001 |  | <0.001 |  | 0.010 |  |
| Per 1-point increase | 2999 | 0.99(0.99,0.99) | **<0.001***** | 0.99(0.99,1.00) | **<0.001***** | 1.00(0.99,1.00) | **0.007**** |

Abbreviations: HR, hazard ratio; CI, confidence interval; CVH, cardiovascular health; LE8, Life’s Essential 8; PAF, population attributable fraction.

All models were stratified by age (>55 and <=55).

Model 1: adjusted for sex, ethnicity;

Model 2: adjusted for sex, ethnicity, Townsend deprivation index, education, income;

Model 3: adjusted for sex, ethnicity, Townsend deprivation index, education, income, cardiovascular disease, cancer, other serious disease.

*P<0.05, **P<0.01, ***P<0.001.

# Supplementary Table 10. Association between healthy lifestyle categories ( poor, intermediate, ideal), Life’s essential 8 (LE8) categories (low, moderate, and high CVH) with the risk of migraine. (excluded other headaches)

|  | Events (%) | Model 1 |  | Model 2 |  | Model 3 |  |
| --- | --- | --- | --- | --- | --- | --- | --- |
|  |  | HR (95%CI) | *P* value | HR (95%CI) | *P* value | HR (95%CI) | *P* value |
| **Healthy lifestyle** | |  |  |  |  |  |  |
| Poor | 1196 (38.2) | 1(reference) |  | 1(reference) |  | 1(reference) |  |
| Intermediate | 1466 (46.8) | 0.85(0.79,0.92) | **<0.001***** | 0.88(0.81,0.95) | **0.002**** | 0.92(0.85,0.99) | **0.048*** |
| Ideal | 472 (15.0) | 0.72(0.65,0.80) | **<0.001***** | 0.76(0.69,0.85) | **<0.001***** | 0.83(0.74,0.92) | **0.001**** |
| *P* for trend |  | <0.001 |  | <0.001 |  | 0.001 |  |
| Per 1-point increase | 3134 | 0.92(0.90,0.95) | **<0.001***** | 0.94(0.92,0.96) | **<0.001***** | 0.96(0.93,0.98) | **0.002**** |
| **CVH categories** | |  |  |  |  |  |  |
| Low CVH | 578 (18.4) | 1(reference) |  | 1(reference) |  | 1(reference) |  |
| Moderate CVH | 2469 (78.8) | 0.84(0.77,0.92) | **<0.001***** | 0.87(0.79,0.95) | **0.003**** | 0.93(0.85,1.02) | 0.209 |
| High CVH | 87 (2.8) | 0.66(0.53,0.83) | **<0.001***** | 0.66(0.53,0.83) | **0.001**** | 0.74(0.59,0.93) | **0.017*** |
| *P* for trend |  | <0.001 |  | <0.001 |  | 0.010 |  |
| Per 1-point increase | 3134 | 0.99(0.99,0.99) | **<0.001***** | 0.99(0.99,1.00) | **<0.001***** | 1.00(0.99,1.00) | **0.007**** |

Abbreviations: HR, hazard ratio; CI, confidence interval; CVH, cardiovascular health; LE8, Life’s Essential 8; PAF, population attributable fraction.

All models were stratified by age (>55 and <=55).

Model 1: adjusted for sex, ethnicity;

Model 2: adjusted for sex, ethnicity, Townsend deprivation index, education, income;

Model 3: adjusted for sex, ethnicity, Townsend deprivation index, education, income, cardiovascular disease, cancer, other serious disease.

*P<0.05, **P<0.01, ***P<0.001.
